# Impact of clinical decision support on controlled substance prescribing

**DOI:** 10.1186/s12911-023-02314-0

**Published:** 2023-10-20

**Authors:** Rachel B. Seymour, Meghan K. Wally, Joseph R. Hsu, PRIMUM Group, Michael Beuhler, Michael J. Bosse, Michael Gibbs, Christopher Griggs, Steven Jarrett, Daniel Leas, Susan Odum, Tamar Roomian, Michael Runyon, Animita Saha, Sharon Schiro, D. Matthew Sullivan, Brad Watling, Stephen Wyatt, Ziqing Yu

**Affiliations:** 1 Department of Orthopaedic Surgery, Atrium Health Musculoskeletal Institute, 1000 Blythe Boulevard, Charlotte, NC 28203 USA; 2 Atrium Health Musculoskeletal Institute, 2001 Vail Avenue, 6th floor, Charlotte, NC 28207 USA

**Keywords:** Opioids, Clinical decision support, Clinical practice guideline, Implementation, Decision-making

## Abstract

**Background:**

Prescription drug overdose and misuse has reached alarming numbers. A persistent problem in clinical care is lack of easy, immediate access to all relevant information at the actionable time. Prescribers must digest an overwhelming amount of information from each patient’s record as well as remain up-to-date with current evidence to provide optimal care. This study aimed to describe prescriber response to a prospective clinical decision support intervention designed to identify patients at risk of adverse events associated with misuse of prescription opioids/benzodiazepines and promote adherence to clinical practice guidelines.

**Methods:**

This study was conducted at a large multi-center healthcare system, using data from the electronic health record. A prospective observational study was performed as clinical decision support (CDS) interventions were sequentially launched (January 2016–July 2019). All data were captured from the medical record prospectively via the CDS tools implemented. A consecutive series of all patient encounters including an opioid/benzodiazepine prescription were included in this study (*n* = 61,124,172 encounters; *n* = 674,785 patients). Physician response to the CDS interventions was the primary outcome, and it was assessed over time using control charts.

**Results:**

An alert was triggered in 23.5% of encounters with a prescription (*n* = 555,626). The prescriber decision was influenced in 18.1% of these encounters (*n* = 100,301). As the number of risk factors increased, the rate of decision being influenced also increased (*p* = 0.0001). The effect of the alert differed by drug, risk factor, specialty, and facility.

**Conclusion:**

The delivery of evidence-based, patient-specific information had an influence on the final prescription in nearly 1 in 5 encounters. Our intervention was sustained with minimal prescriber fatigue over many years in a large and diverse health system.

**Supplementary Information:**

The online version contains supplementary material available at 10.1186/s12911-023-02314-0.

## Background

Prescription drug overdose and misuse has reached alarming numbers. Increases in opioid prescriptions for acute and chronic pain have played a significant role in the rise of opioid use disorders and overdose [1, 2]. While the most common class of scheduled medications involved in deaths related to pharmaceutical overdose is opioids (75.2%), benzodiazepines are involved in nearly one-third of these overdoses (29.4%) as co-ingestants [3]. Opioids are implicated in 77.2% of deaths involving benzodiazepines, making these two classes of drugs an ideal focus for intervention [3]. There has been an overall decrease in life expectancy since 2014, and opioid-deaths have been associated with a 0.21-year reduction in average life expectancy alone, indicating the extent of their impact [4, 5].

Primary prevention must be at the forefront of solutions to this crisis [6]. Identifying “at risk” patients prior to prescribing is an important step in reducing prescription medication use disorder, diversion, and overdose. According to a recent study by the Centers for Disease Control and Prevention (CDC), 21% of patients who receive their first opioid prescription continue to receive additional prescriptions episodically, and 6% progress to long-term use [7]. In fact, approximately half of patients taking opioids for at least three months remain on opioids after five and are unlikely to stop taking these medications [8–10]. Up to 43% of patients who develop an addiction to prescription opioids originally received the medication from a health care professional [11–14]. Previous research by our team found that 25% of patients presenting to a Level 1 trauma center with orthopaedic injuries had risk factors for misuse prior to the injury. An additional 9% of these patients developed new risk characteristics during the follow-up period [15]. These findings herald an opportunity to involve prescribers in primary prevention and improve patient safety by utilizing real-time decision support.

In 2016, the CDC released a clinical practice guideline (CPG) for opioid prescribing for chronic pain that provides information regarding: 1) determining when to initiate or continue opioids for chronic pain; 2) opioid selection, dosage, duration, follow-up, and discontinuation; and 3) assessing risk and addressing harms of opioid use [16]. In North Carolina, the Strengthening Opioid Misuse Prevention (STOP) Act of 2017 limits the duration of prescriptions for acute and post-operative pain to 5 or 7 days, respectively [17]. While the goal of prescribers is to manage patient’s pain while optimizing patient safety and following current guidelines, few strategies have been implemented and evaluated to support prescribers in this effort at the point of care.

A persistent problem in clinical care is lack of easy, immediate access to all relevant information at the moment it is needed and actionable. Prescribers must digest an overwhelming amount of information from each patient’s electronic health record (EHR) as well as remain up-to-date with knowledge of current evidence to provide optimal care. The Prescription Reporting with Immediate Medication Utilization Mapping (PRIMUM) is integrated into the existing clinical workflow of clinicians in the EHR and designed to improve patient safety by providing the necessary, relevant, and objective information to prescribers at the point of care via an alert to identify patients at risk for opiate misuse disorder, overdose, and the diversion of prescription opioids. Additionally, this work provided a pathway to analyzing the data on a continuous basis and allowed our team to refine the alert based on prescriber feedback [15, 18]. Finally, the original clinical decision support (CDS) tool provided a platform from which to build and operationalize the CDC CPG to promote adherence to best practices. This platform provides the foundation for a continually evolving prospective intervention that can be modified in response to new evidence to support optimal clinical decision-making.

In summary, the purpose of the PRIMUM [18] project was to:

1. Leverage the power of the EHR to describe the pattern of opioid and benzodiazepine prescribing across our large health system.
2. Implement prescriber alerts based on peer-reviewed literature and consensus opinion that capitalize on searchable, objective EHR data to identify at-risk patients at the point of prescription.
3. Operationalize the CDC Guideline for Prescribing Opioids for Chronic Pain within the EHR.

This study aimed to evaluate the impact of these sequentially launched CDS tools on opioid and benzodiazepine prescribing behavior, over time. In addition, this study aimed to determine whether specific patient risk factors, prescriber specialty, prescriber type, and facility type were associated with prescriber response to the CDS tools.

## Methods

### Intervention

Following IRB approval (including approval of waived informed consent), a multidisciplinary expert panel (clinicians and researchers from orthopaedic surgery, emergency medicine, internal medicine, family medicine, addiction medicine, pharmacy, administration, information and analytic services, and public health) convened to create an EHR intervention as part of a strategy to address the opioid crisis and patient safety. The central EHR (Cerner®) is utilized in 20 hospitals and emergency departments, 450 + outpatient specialty and primary care clinics, and 30 + urgent care facilities throughout our system. The team identified the following risk factors for misuse, abuse, or diversion of opioids or benzodiazepines through literature reviews:

1. “early refill,” defined as current prescription with >50% remaining [19];
2. two or more emergency department (ED) or Urgent Care visits with on-site opioid administration within the previous 30 days [20–22];
3. three or more prescriptions for opioids, benzodiazepines, or both within the previous 30 days [19, 20, 23–25];
4. previous opioid or benzodiazepine overdose [26]; and
5. positive toxicology screen for blood alcohol, cocaine, or marijuana in the EHR [27–31].

These risk factors were built as triggers for a rule within the EHR that screens each opioid or benzodiazepine prescription and powers a prescriber-facing alert when patients have at least one risk factor. Upon receiving the alert, prescribers have the option to either continue or cancel the prescription. This EHR alert went live as part of a phased roll-out during 2015.

In 2017, following the release of the CDC Guideline, CDS interventions were added to the PRIMUM platform to operationalize the CDC CPG into the EHR workflow. First, we built a controlled substances review component within the EHR that concatenates and displays all of the risk factors, external links to our local prescription drug management programs (PDMPs), internal hyperlink to view the patient’s current pain agreement or complete one, all controlled substances prescribed or administered, and morphine milligram equivalent (MME) information for active prescriptions Second, additional triggers were added to the existing PRIMUM alert to notify the prescriber of co-prescribing (when a prescriber was prescribing a benzodiazepine for a patient already receiving an opioid or vice versa). Finally, three new alerts were developed: 1) an alert if an extended release opioid is prescribed for an opioid naïve patient; 2) an alert that fires when patients reach 90 days of continuous opioid therapy to suggest initiation of a pain agreement and regular urine drug screens; and 3) an alert suggesting prescribing naloxone for patients at high risk of overdose (defined as co-prescribed opioids and benzodiazepines, MME > 50, history of overdose). In addition to these alerts, we developed a standard electronic pain agreement, standard patient education materials automatically printed for patients prescribed an opioid, and an educational webpage for clinicians.

### Data collection

The CDS rule automatically generates regular reports of every opioid or benzodiazepine prescription written in the healthcare system, including information about the patient, the prescriber, the facility, and the drug.

Ideally, the alert and data collection would occur once the prescription is complete, allowing capture of full prescription details and prescriber response. Focus group data indicated an alert at this point would be too late and disrupt workflow. Clinicians would be faced with restarting the entire prescription or ignoring the intervention. To bring the intervention closer to the decision point, we chose to fire the logic as soon as the medication was selected. This decision optimized the clinical efficiency of the intervention to gain prescriber acceptance and prevent alert fatigue.

To determine if the alerts influenced prescribing behavior, we calculated rates of prescribers choosing the “cancel” and/or “continue” option upon receiving an alert. If a prescriber received an alert and chose the “cancel” option, we considered the alert to have influenced their prescribing decision (referred to as “decision influenced” hereafter). After clicking the “cancel” option, the prescriber may have initiated a different prescription (and received another alert) which was completed (i.e. after discussing with the patient, further chart review, etc.). These are classified as “cancelled” in the results to indicate the initial prescription was cancelled. Due to the limited data capture of canceled prescriptions because of the timing of the alert described above, we are unable to quantify or describe the exact influence on the completed prescription. Alternatively, the prescriber may have decided not to prescribe opioids or benzodiazepines at all after clicking “cancel”. In this case, the patient was considered to have “none ordered”. Therefore, rates of “none ordered” and “cancelled” are mutually exclusive and exhaustive subsets of the reported “decision influenced” rates. Our primary outcome measure was “decision influenced”, because selecting ‘cancel’ indicates a change in prescriber behavior in response to the alert. Average total MME dose and daily MME [32] were calculated, and the duration of prescription was obtained.

### Statistical analysis

Descriptive statistics were used to characterize opioid prescribing. We calculated the incidence of “decision influenced” in response to alerts over time and compared these rates by number of risk factors, medication type, specialty, facility, and prescriber type using chi-squared tests. Finally, we assessed response to the 90-day alert trigger by calculating the percentage who initiated a pain agreement after receiving this alert. Similarly, we assessed response to the naloxone alert by calculating the percentage who prescribed naloxone after receiving this alert.

Given the multifactorial influences that could have occurred concurrently during the implementation of the alert system (i.e. educational programs, legislation, media reports relating to opioid or benzodiazepine prescribing), Shewhart p-charts (a.k.a. control charts) were used to evaluate binary outcomes longitudinally. Control charts are a statistical tool commonly used in quality improvement to evaluate process stability over time(i.e., the CDS process and its influence on the prescribers’ decision to prescribe opioids). The frequency of the decision influenced was aggregated by month and January 1, 2016-December 31, 2016 was considered the baseline period (i.e., after the initial CDS alert was launched, but before subsequent rollout of additional CDS tools). The control chart was created by plotting the monthly average number of times the decision to prescribe opioids was influenced by the CDS with control limit lines marked by one sigma, twos sigma and three sigmas above and below the average. Longitudinal change in the pattern of the decision influenced was evaluated for common cause and special cause variation [33]. Common cause variation indicates that a process is stable, or under control, as the variation in the data is predictable and expected with a random pattern when graphically displayed. Common cause is inherent in the system; therefore, it does not affect quality or require process improvements. Special cause variation occurs when data varies in an unexpected, non-random pattern indicating an unstable process. Special cause variation indicates major shifts in a process that warrant evaluation to determine the sources and impacts of any system changes. Specifically, we expected to see improved rates of prescribers altering opioid prescriptions when the CDS alert was present as each incremental change to the CDS was implemented. Evidence for special cause variation was assessed according to rules set by the Institute for Healthcare Improvement (IHI). [34, 35].

## Results

Throughout the entire evaluation period (January 1, 2016 – July 31, 2019), our decision support tool was applied to 61,124,172 encounters in our healthcare system. Of these, 2,368,118 (3.9%) included a prescription opioid or benzodiazepine, representing 674,785 unique patients. A PRIMUM alert was triggered in 23.5% of these encounters.

Characteristics of the prescribing encounters (encounters resulting in a completed opioid or benzodiazepine prescription) are presented in Table 1. Prescriptions were most often written to patients ages 18–64 (70.1%) and in outpatient clinics (68.4%). Physicians wrote the majority of prescriptions (65.5%), and primary care prescribers wrote the most (42.5%), followed by emergency or urgent care prescribers (20.2%). Opioids were prescribed more frequently than benzodiazepines (75.6% and 21.6%, respectively).

**Table 1:** Characteristics of prescribing encounters (*n* = 2,321,059)

	**#**	**%**
**Age of Patient**		
**< 18 years**	51,856	2.2
**18–64**	1,626,021	70.1
**≥ 65**	643,182	27.7
**Facility Type**		
**ED/Urgent Care**	529,270	22.8
**Inpatient Discharge**	179,705	7.7
**Other**	24,132	1.0
**Outpatient**	1,587,952	68.4
**Prescriber Type**		
**Physician**	1,520,158	65.5
**Physician Assistant**	439,310	18.9
**Nurse Practitioner**	338,762	14.6
**Other/Unknown**	22,829	1.0
**Class of Drug**		
**Opioid**	1,755,360	75.6
**Benzodiazepine**	500,211	21.6
**Both**	65,487	2.8
**Number of Criteria Met (*n* = 997,518)**^a^		
**0**	730,763	73.3
**1**	194,104	19.5
**2**	57,671	5.8
**3**	13,479	1.4
**≥4**	1,501	0.2
**Category of Criteria Met (*n* = 997,518)**^a^		
**Prescription with > 50% remaining (“early refill”)**	83,697	8.4
**2 + visits with Onsite Administration**	12,071	1.2
**3 + Prescriptions in past 30 Days**	49,644	5.0
**Positive Tox (any)**	58,272	5.8
**Previous Presentation for Overdose**	10,532	1.1
**Co-prescribed Benzos and Opioids**	138,092	13.8
**Extended Release**	3,538	0.4
**90-day**	119,153	11.9
**Naloxone**	105,817	10.6
**Medical Specialty**		
**Behavioral Health**	44,288	1.9
**Cancer**	55,847	2.4
**Emergency/Urgent Care**	469,110	20.2
**Pain**	303,130	13.1
**Primary Care**	986,039	42.5
**Medical Specialty**	86,864	3.7
**Surgical Specialty**	275,503	11.9
**Long Term Care**	900	0.04
**Unknown**	99,378	4.3
**Duration**		
**< 3 days**	470,890	20.3
**4–7 days**	426,805	18.4
**8–14 days**	257,239	11.1
**15–29 days**	167,505	7.2
**30–59 days**	900,116	38.8
**60 + days**	98,502	4.2
**MME (*n* = 1,820,848)**^b^		
**< 50**	1,390,514	76.4
**50–89**	255,987	14.1
**90 + **	174,347	9.6

*ED* Emergency department, *tox* Toxicology, *MME* Morphine milligram equivalent

^a^Since additional triggers were added to the alert during the study period, these were only described for the time period during which all interventions were live (10/17/2017–07/31/2019)

^b^MME excludes benzodiazepine prescriptions and records where MME = 0 or where MME was missing

Patient risk factors were assessed during the time frame in which all intervention components were active to allow for comparison (October 17, 2017 – July 31, 2019). While most patients had no risk factors (73.3%), of those with risk factors, most only had one (75.4%). Co-prescription of benzodiazepines and opioids was the most prevalent trigger (13.8%), followed by “early refill” (8.4%). 11.9% of patients triggered the 90-day alert, and 10.6% of patients triggered the naloxone alert. Most patients had daily MME below 50 (76.4%), and most prescriptions were written for ≤ 7 days (38.7%) or between 30–59 days (38.8%).

**Fig. 1:**
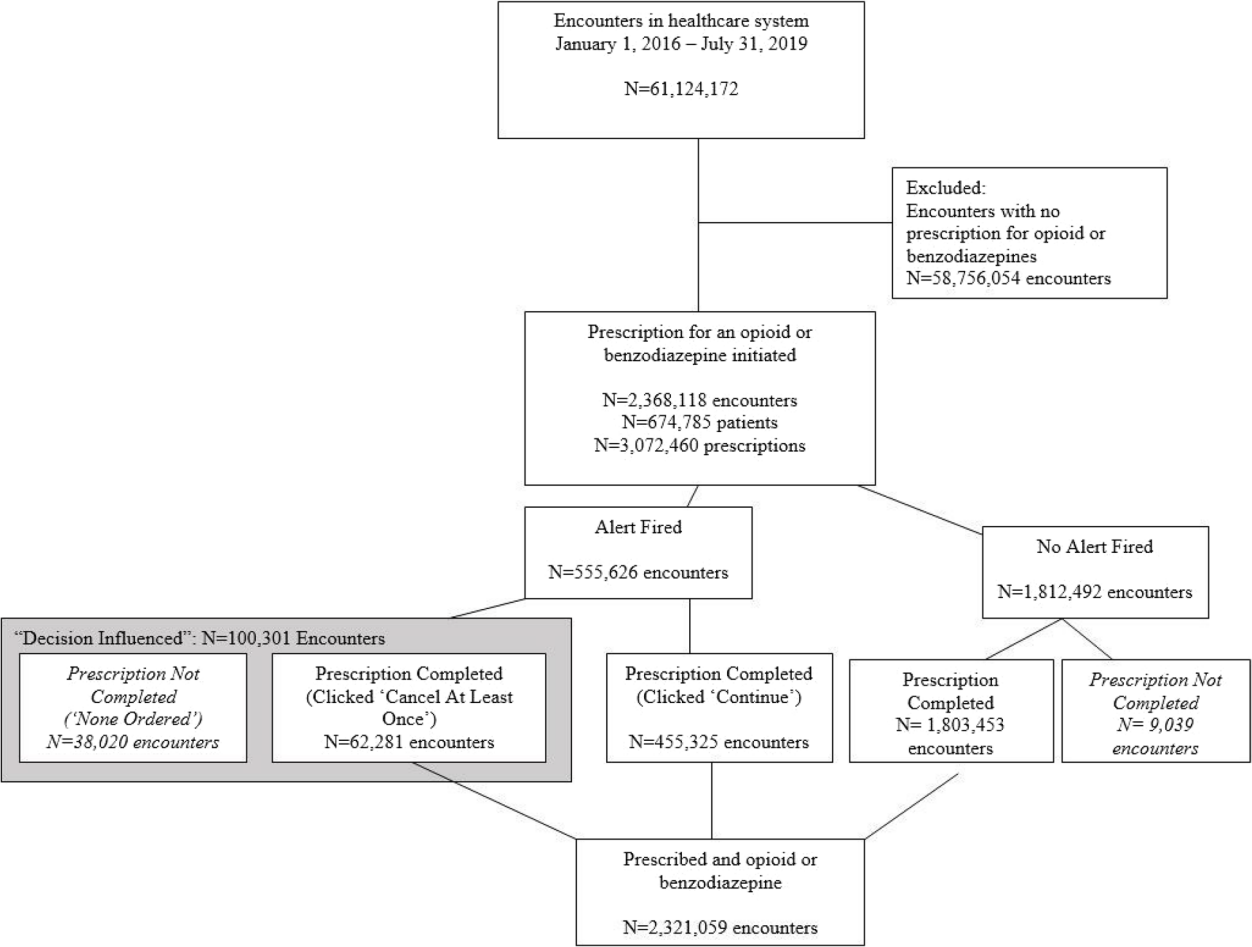
Prescription flow diagram

**Fig. 2:**
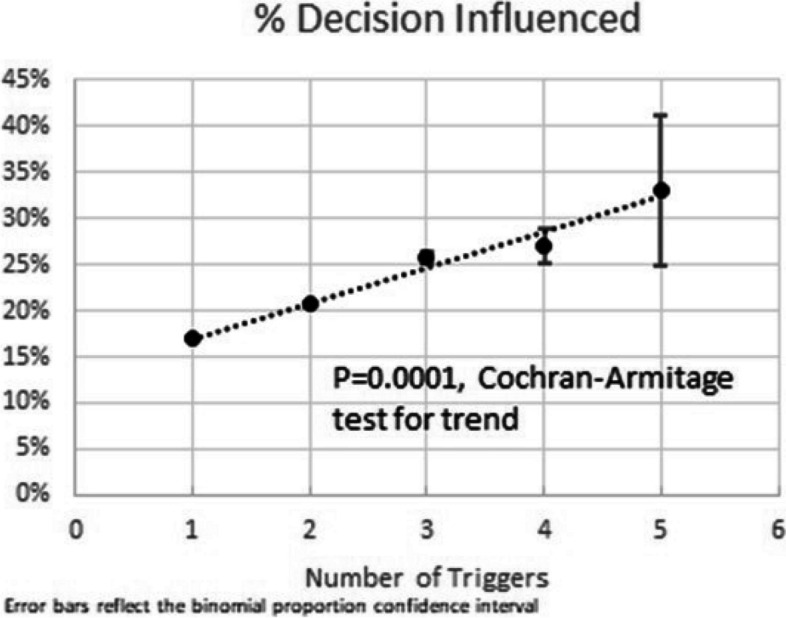
Association between number of triggers and “decision influenced”

The logic screened over 61 million encounters, of which 555,626 triggered an alert (23.5% of encounters with a prescription) (Table 2; Fig. 1). The prescriber decision was influenced in 18.1% of these (*n* = 100,301). The rate of “decision influenced” increased as the number of risk factors increased (Cochran-Armitage test for trend, *p* = 0.0001; Fig. 2). Alerting had a greater influence on benzodiazepines (25.6%) as compared to opioids (15.7%) (*p* < 0.001). The trigger with the largest “decision influenced” rate was the extended release alert (24.3%), followed by “early refill” (23.2%). Onsite administration of opioids or benzodiazepines at least twice within the past 30 days had the lowest rate of “decision influenced” (13.5%), followed by positive toxicology screen (14.7%). The rate of “decision influenced” also varied by prescriber specialty (*p* < 0.001) and facility type (*p* < 0.0001). Long-term care and cancer prescribers had the highest rates (35.1% and 24.7%, respectively), while rates were lowest in emergency medicine and pain specialties (11.1% and 12.0%, respectively). Prescribers in the inpatient setting had the highest rate of “decision influenced” (23.3%), while rates were lowest in ED and Urgent Care setting (11.6%). The response to the 90-day and naloxone alerts was low. Only 3.1% initiated a pain agreement (*n* = 3,634), and only 2.3% prescribed naloxone (*n* = 2,463). While the percentages were low, these represented thousands of interventions each.

**Table 2:** Rates of decision influenced by trigger, alert, and medication type, among encounters with an alert, January 2016–July 2019

	Number of Encounters	N Decision Influenced^a^	% Decision Influenced*
**Prescription Alert**			
All Encounters with Alert	555,626	100,301	18.1
Number of Triggers*			
1	419,088	70,825	16.9
2	113,887	23,603	20.7
3	20,378	5,249	25.8
4	2,135	579	27.1
5	130	43	33.1
6	8	2	25.0
Medication Type*			
Opioid	412,189	64,782	15.7
Benzo	106,468	27,268	25.6
Both	36,866	8,148	22.1
PRIMUM Alert Trigger			
Early Refill	229,250	53,105	23.2
Positive Tox	148,968	21,936	14.7
≥3 Prescriptions	126,568	25,917	20.5
≥2 Onsite Administrations	34,794	4,681	13.5
History of Overdose	25,210	4,850	19.2
Co-prescribed Opioid and Benzo Extended Release	148,103 4,142	24,755 1,007	16.7 24.3
Specialty*			
Behavioral Health	13,222	2,588	19.6
Cancer	21,596	5,334	24.7
Emergency	88,698	9,879	11.1
Long Term Care	331	116	35.1
Pain	94,143	11,248	12.0
Primary Care	249,611	554,625	21.9
Medical Specialty	21,313	4,451	20.9
Surgical Specialty	46,428	7,208	15.5
Unknown	20,284	4,852	23.9
Facility Type*			
ED/Urgent Care	98,391	11,382	11.6
Inpatient	37,757	8,785	23.3
Outpatient	411,256	77,772	18.9
Other	8,222	2,362	28.7
Prescriber Type*			
Physician	359,913	57,711	16.0
Physician Assistant	93,525	12,354	13.2
Nurse Practitioner	82,014	12,679	15.5
Other/Unknown	20,174	17,557	87.0
**90-Day Alert**^b^	119,153	3,634	3.1
**Naloxone Alert**^b^	105,817	2,463	2.3

^*^P < 0.0001, Chi-square

^a^Decision Influenced for prescription alert is clicking “cancel” in response to the alert; for 90 day alert it is initiation of pain agreement; for naloxone alert it is prescription of naloxone

^b^These data only represent the period of time during which these interventions were live: October 2018-July 2019

**Fig. 3:**
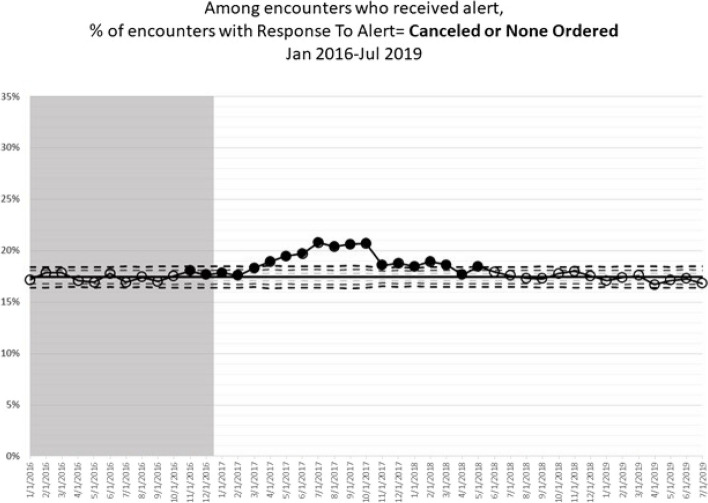
Percent of encounters including an alert where prescribing decision was influenced, Over Time. “Decision Influenced” was defined as the prescriber clicking “cancel” when they saw the CDS alert at least once during the encounter. This includes cases where they ultimately prescribed an opioid or benzodiazepine later in the same encounter *and* cases where no opioid or benzodiazepine was prescribed. Filled points represent evidence for special cause variation

**Fig. 4:**
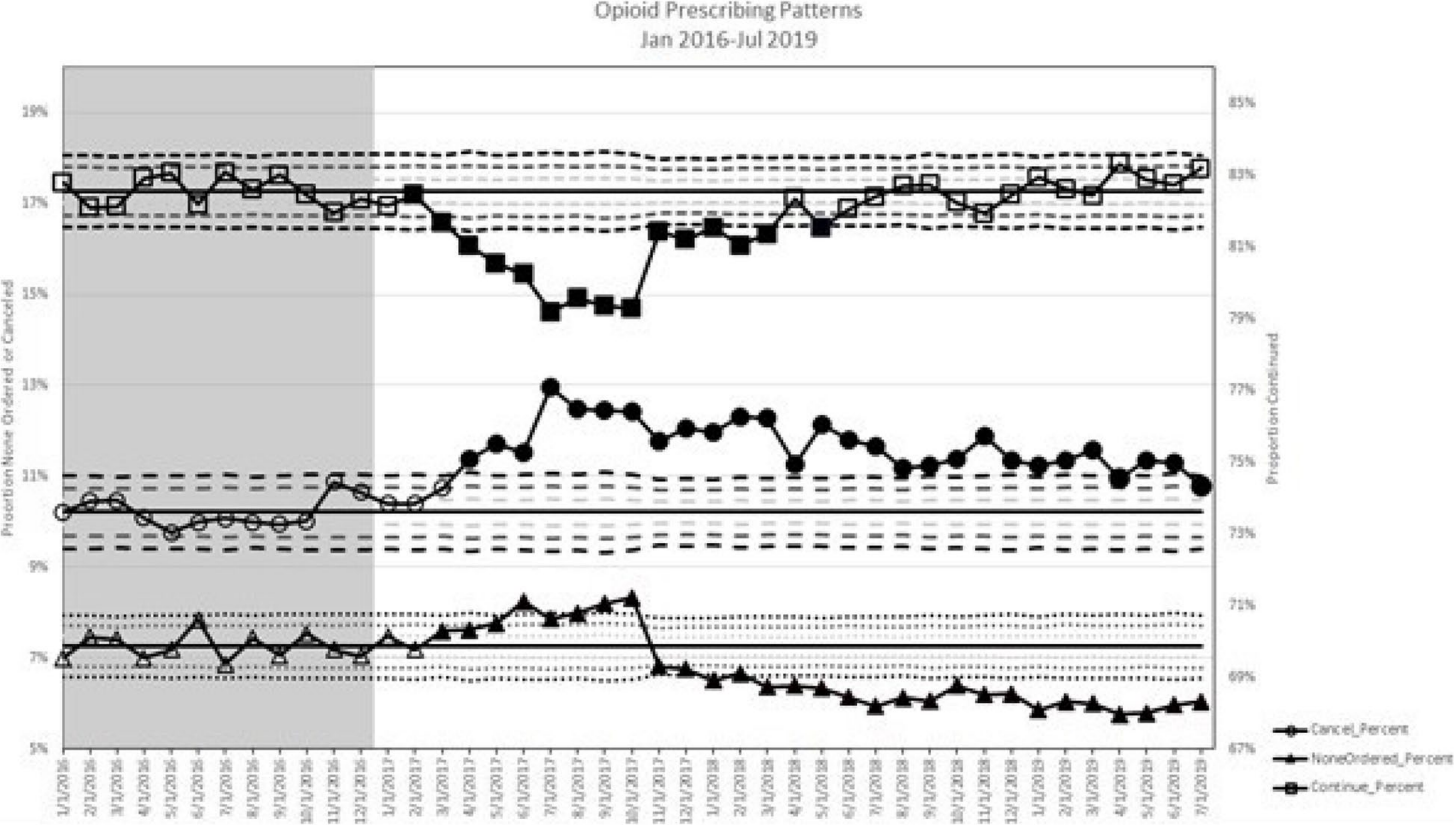
Rate of prescriptions continued, cancelled, and not ordered over time. Continued indicates a prescriber clicked “continue” after receiving the alert. Cancelled means the prescriber clicked “cancel”, then ultimately prescribed an opioid or benzodiazepine during that encounter. None Ordered means the prescriber clicked “cancel” and did *not* prescribe an opioid or benzodiazepine during that encounter. Filled points represent evidence for special cause variation

Figures 3 and 4 show the rates of “decision influenced” over time, using 2016 as baseline. Filled symbols represent evidence for special cause variation, indicating that the intervention is responsible for the change in prescribing behavior as opposed to common cause, or natural variation. Overall, the rate of “decision influenced” consistently increased as compared to baseline from the beginning of 2017 through May of 2018. The controlled substance review component was launched in March of 2017, and the rest of the CDC Guideline Implementation interventions were launched in October of 2017. Therefore, the change does not align with the launch of specific interventions, and is likely due to other concurrent factors (i.e. increased awareness of opioid prescribing over time, department-specific initiatives, state-wide educational requirements). While the rates of “decision influenced” did return to rates similar to baseline after May of 2018, there was not a consistent downward trend. The rates of “none ordered” fell substantially in November of 2017, which aligns with the launch of the CDC Guideline operationalization components (see Supplemental Material). At the same time, the rates of cancellations remained increased from March of 2017 throughout the study period relative to baseline.

## Discussion

Our team built an EHR-integrated CDS platform to address opioid and benzodiazepine prescribing by providing critical, evidence-based, objective information at the point of care. The goal of this intervention was to provide information within the workflow to support clinical decision-making, increase patient safety, and decrease subjectivity when assessing risk for prescription drug misuse. We believe the CDS is useful to the prescriber, as indicated by the number of prescriptions modified. We further believe the act of clicking ‘cancel’ likely signals the prescriber’s desire to more carefully consider the appropriateness of the prescription in light of patient risk factors. Most encounters where the alert was initially cancelled ultimately resulted in a prescription. It is possible the alert influenced prescriber behavior resulting in a prescription of a different drug, dose, or duration than originally intended; however, our methodology did not allow for a full exploration of this hypothesis since we prioritized the efficiency and durability of the clinical intervention. While most encounters resulted in a prescription, high rates of influence were demonstrated (*n* = 100,301), and cancellation (“none ordered”) of *n* = 38,020 prescriptions resulted from delivering risk information during the prescribing process.

The rates of “none ordered” decreased with the launch of the CDC Guidelines CDS tools, while the rate of cancellations remained at an increased rate as compared to baseline. This suggests our alert has had a consistently increased impact on opioid prescribing, despite the fact that patients continue to receive controlled substances. It is possible the operationalization of the CDC Guidelines promoted safe and appropriate prescribing rather than avoidance of prescribing altogether. Further, our educational efforts have focused on balancing patient comfort and safety as opposed to simple opioid restriction. In addition, safe prescribing practices may increase the number of prescriptions written by replacing prescriptions of longer durations with more prescriptions of shorter duration each. These findings are similar to a study assessing an opioid CDS intervention which found improvement in adherence to guidelines and safe opioid prescribing (i.e., provision of naloxone and opioid treatment agreements), without any change in prescribing outcomes such as number of opioid prescriptions or opioid overdose [36]. In fact, this study found a “worsening” trend in the rate of opioid prescribing after launch of the CDS intervention, similar to our finding of lower rates of “none ordered”. Outside the scope of a CDS intervention, overall, release of the CDC guideline was associated with reduction in duration of opioid prescriptions and reduction of high-dose prescribing, without an impact on initiation of opioid therapy [37]. We believe our data supports maturation of this balanced approach to opioid prescribing. However, there are also examples in the literature of CDS interventions that improve adherence to guidelines and decreased rates of opioid prescribing overall [38].

This intervention was implemented during a time of drastic increase in clinical and lay awareness about the opioid crisis, so we could not determine the unique contribution of the intervention on prescribing rates. Thus, our focus on “decision influenced” was most appropriate because it is the most proximal behavior to our alert, and we cannot expect this intervention to affect prescribing behavior in encounters which do not trigger an alert. Additionally, our healthcare system grew throughout the study period with the addition of new practices and prescribers, further complicating analysis of prescribing rates. Particularly, our system transitioned many pain clinics onto our common EHR throughout our study period.

Another limitation is the specific choice of risk factors that triggered the prescriber alerts. While every effort was made to utilize objective, evidence-based choices, other risk factors might improve sensitivity to identify “at risk patients”. However, this does not detract from the overall purpose of this large-scale intervention which was to demonstrate the effectiveness of presenting relevant clinical information to prescribers in real time to allow for modification of prescribing practices.

A large proportion of patients with risk factors continued to receive prescriptions following an alert, indicating that prescriber decision-making is complex and multifactorial. As there are legitimate medical uses for these medications, the desired outcome is not always preventing a prescription. While many of these prescriptions might be medically indicated and additional subgroup analysis may provide better understanding of prescribing patterns, there is likely still room for improvement. Additional analysis is needed to fully understand the effect of this alerting across specialties, facilities, prescribers, and patient subgroups. Further exploration of these groups will be conducted in order to inform the need for more targeted interventions.

The PRIMUM platform presents an opportunity to utilize the EHR across millions of encounters to provide targeted CDS around appropriate prescribing, which is a worthwhile and important effort [39]. The platform is dynamic, allowing for iterative improvement and responsiveness to prescriber feedback and new evidence. It also lays the groundwork for the addition of new CDS tools. Future work could include electronic protocols for tapering, a streamlined method for checking PDMPs or otherwise assess existing prescriptions prior to prescribing, inclusion of alternative modalities for the treatment of pain, and targeted interventions for specific specialties or facilities.

## Conclusion

In conclusion, nearly 20% of prescriptions were influenced by delivering evidence-based, patient-specific information during the prescription ordering process. Our intervention influenced a large percentage of prescriptions and was sustained without fatigue over many years in a large system of diverse specialties and facilities.

### Supplementary Information

**Additional file1:** **Supplemental Materials Table S1.** Operationalization of the CDC Guideline Recommendations into the Electronic Health Record. **Figure S1.** Prescription Narcotic Alert. **Figure S2.** Controlled Substance Review. **Figure S3.** Opioid 90 Day Therapy Alert. **Figure S4.** Prescribe Naloxone Alert.

## Data Availability

The datasets used and/or analyzed during the current study are available from the corresponding author on reasonable request.
