# Supplementary material for: Impact of clinical decision support on controlled substance prescribing
Source: BMC Med Inform Decis Mak. 2023 Oct 20;23:234. doi: 10.1186/s12911-023-02314-0 (PMC10588193; doi:10.1186/s12911-023-02314-0)

Supplemental Materials

TableS1: Operationalization of the CDC Guideline Recommendations into the Electronic Health Record

| **Recommendation Statement​** | **Intervention Component​** |
| --- | --- |
| 1. Non-pharmacologic therapy and non-opioid pharmacologic therapy preferred.​ | Education​ |
| 2. Establish treatment goals and set expectations.​ | Standard pain agreement and prompt to complete at 90 days of opioid therapy​ |
| 3. Discuss risks and benefits of opioids.​ | Standard patient education printed with each prescription​ |
| 4. Start with immediate-release opioids. ​ | Alert when ER/LA opioid selected for opioid naïve patient prompting immediate release instead.​ |
| 5. Prescribe lowest effective dose. Use cation above 50 and/or 90 MME/day​ | Controlled Substance Review Component displays total MME. Alert if prescribing >50 or >90. ​ |
| 6. Lowest effective dose, 3-7 days for acute pain.​ | Education, acute prescribing policy in progress​ |
| 7. Evaluate within 1-4 weeks after starting and every 3 months after.​ | Reminder alert of guideline when patient reaches 90 days of opioids​ |
| 8. Consider offering naloxone when factors increase risk. | Alert to prescribe naloxone if history of overdose, above 50 MME, or current benzodiazepine |
| 9. Clinicians should review PDMP. | Hyperlink to PDMP on Controlled Substance Review Component |
| 10. Clinicians should use urine drug screens | Alert suggesting urine drug testing at 90 days of opioid therapy. |
| 11. Clinicians should avoid prescribing opioids and benzodiazepines concurrently. | Alert to indicate co-prescription of opioids and benzodiazepines. |
| 12. Clinicians should offer or arrange evidence-based treatment for patients with opioid use disorder. | Education |

Figure S1: Prescription Narcotic Alert


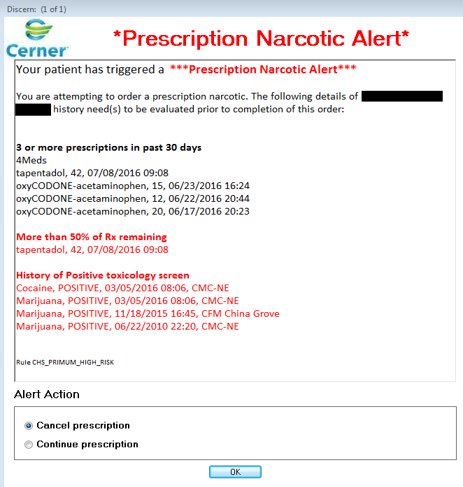


Figure S2: Controlled Substance Review

 
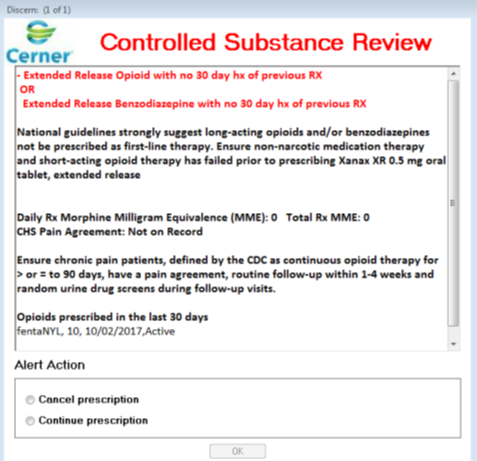


Figure S3: Opioid 90 Day Therapy Alert

 
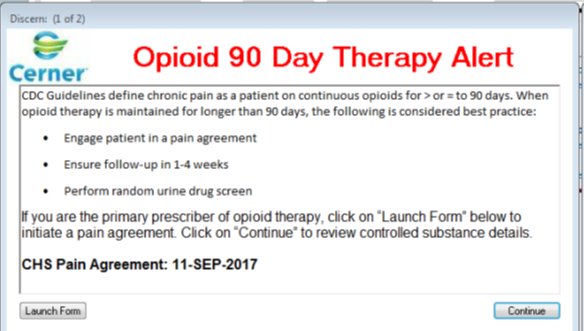


Figure S4: Prescribe Naloxone Alert

 
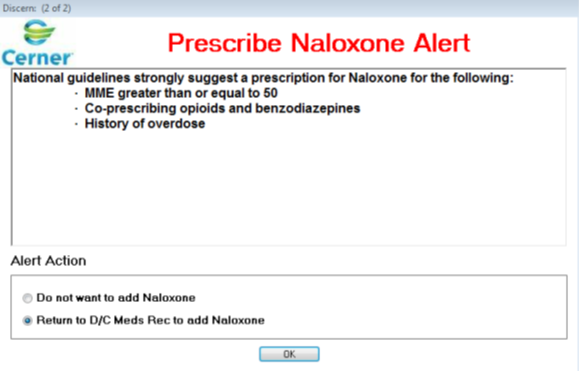

Supplement: Supplementary file 1 — Additional file1: Supplemental Materials Table S1. Operationalization of the CDC Guideline Recommendations into the Electronic Health Record. Figure S1. Prescription Narcotic Alert. Figure S2. Controlled Substance Review. Figure S3. Opioid 90 Day Therapy Alert. Figure S4. Prescribe Naloxone Alert. [file 12911_2023_2314_MOESM1_ESM.docx]
